# Association of Difference Between eGFR From Cystatin C and Creatinine and Serum GDF‐15 With Adverse Outcomes in Diabetes Mellitus

**DOI:** 10.1002/jcsm.70011

**Published:** 2025-07-23

**Authors:** Tomohito Gohda, Nozomu Kamei, Marenao Tanaka, Masato Furuhashi, Tatsuya Sato, Mitsunobu Kubota, Michiyoshi Sanuki, Takeo Koshida, Shinji Hagiwara, Yusuke Suzuki, Maki Murakoshi

**Affiliations:** ^1^ Department of Nephrology Juntendo University Faculty of Medicine Tokyo Japan; ^2^ Department of Endocrinology and Metabolism Hiroshima Red Cross Hospital & Atomic‐Bomb Survivors Hospital Hiroshima Japan; ^3^ Institute for Clinical Research, NHO Kure Medical Center and Chugoku Cancer Center Hiroshima Japan; ^4^ Department of Cardiovascular, Renal and Metabolic Medicine Sapporo Medical University School of Medicine Sapporo Japan; ^5^ Department of Cellular Physiology and Signal Transduction Sapporo Medical University School of Medicine Sapporo Japan; ^6^ Department of Endocrinology and Diabetology NHO Kure Medical Center and Chugoku Cancer Center Hiroshima Japan

**Keywords:** chronic kidney disease, diabetes mellitus, difference between estimated glomerular filtration rates derived from cystatin C and creatinine (eGFRdiff), growth differentiation factor‐15, mortality, sarcopenia

## Abstract

**Background:**

Protein catabolism and chronic inflammation drive sarcopenia and frailty in individuals with diabetes mellitus and chronic kidney disease (CKD). The difference between estimated glomerular filtration rates derived from cystatin C and creatinine (eGFRcys and eGFRcr, respectively), termed eGFRdiff, along with growth differentiation factor‐15 (GDF‐15) levels, have emerged as markers of metabolic and inflammatory dysregulation. Lower eGFRdiff and elevated GDF‐15 levels are associated with sarcopenia, frailty, CKD progression and mortality. However, their interplay and respective impacts on CKD progression and mortality remain unclear.

**Methods:**

A total of 638 Japanese individuals with diabetes mellitus were stratified into tertiles based on eGFRdiff. Serum GDF‐15 levels were measured using enzyme‐linked immunosorbent assays. The relationships between eGFRdiff and GDF‐15 were assessed using Spearman's correlation coefficients. Multivariate ordered logistic regression was used to evaluate the association between eGFRdiff and GDF‐15 tertiles, with GDF‐15 as the dependent variable and eGFRdiff as the independent variable, adjusting for covariates including age, sex, urinary albumin‐to‐creatinine ratio (UACR) and eGFRcr or eGFRcys. Cox proportional hazards models with restricted cubic splines were used to examine associations between eGFRdiff and GDF‐15 (independent variables) with CKD progression (≥ 30% decline in eGFRcr from baseline) and mortality (dependent variables). These models were adjusted for age, sex, glycated haemoglobin, UACR and eGFRcr.

**Results:**

The median age was 65 years (interquartile range: 58–73), and 53.9% of participants were male. Over median follow‐up periods of 5.3 years for CKD progression and 5.4 years for mortality, 75 participants (11.8%) experienced CKD progression and 44 (6.9%) died. GDF‐15 levels inversely correlated with eGFRdiff (*r* = −0.35, *p* < 0.001). Higher eGFRdiff values were associated with lower odds of being in a higher GDF‐15 tertile (odds ratio 0.86; 95% confidence interval [CI]: 0.76–0.97; *p* = 0.01). Both lower eGFRdiff and higher GDF‐15 levels were independently associated with adverse outcomes: CKD progression (GDF‐15, hazard ratio [HR] 1.36, 95% CI: 1.02–1.81, *p* < 0.05; eGFRdiff, HR 0.67, 95% CI: 0.58–0.78, *p* < 0.0001) and mortality (GDF‐15, HR 2.35, 95% CI: 1.63–3.41, *p* < 0.0001; eGFRdiff: 0.80, 95% CI: 0.65–0.99, *p* < 0.05).

**Conclusions:**

Both eGFRdiff and GDF‐15 were independently associated with adverse outcomes in individuals with diabetes mellitus. GDF‐15 showed a stronger association with mortality, whereas eGFRdiff was more strongly linked to CKD progression. These findings underscore the potential utility of these markers in risk stratification for diabetes‐related complications and may guide individualized interventions in clinical practice.

## 1 Introduction

As chronic kidney disease (CKD) progresses, chronic inflammation and metabolic disturbances contribute to muscle loss, thereby increasing the risk of sarcopenia [1]. Sarcopenia is not merely a consequence of CKD but may also be a driver of its progression [2, 3]. In older adults or individuals with frailty and sarcopenia, reduced muscle mass leads to decreased serum creatinine levels—reflecting diminished muscle creatine metabolism. This reduction can overestimate the creatinine‐based estimated glomerular filtration rate (eGFRcr), even when the actual GFR remains stable. In contrast, cystatin C, a marker less influenced by muscle mass, provides a more accurate estimation of kidney function through cystatin C‐based eGFR (eGFRcys).

The difference between eGFRcys and eGFRcr, termed eGFRdiff, has recently emerged as a clinically relevant parameter. Previous studies have linked eGFRdiff to a range of adverse outcomes, including falls, hospitalization, kidney failure, cardiovascular disease (CVD) and mortality [4, 5, 6, 7, 8]. Matsuzawa et al. [9] further identified five serum indices derived from cystatin C and creatinine—eGFRdiff, sarcopenia index (SI), serum creatinine/cystatin C ratio (CCR), a predictive equation for skeletal muscle mass index (pSMI) and total body muscle mass index (TBMM)—as effective tools for identifying sarcopenia, including in community‐dwelling elderly Japanese populations.

Growth differentiation factor‐15 (GDF‐15), a stress‐responsive cytokine in the transforming growth factor‐β superfamily, is released in response to tissue injury, inflammation, hypoxia and malignancies in various organs [10, 11, 12]. In individuals with type 2 diabetes, CKD and heart failure, GDF‐15 levels are elevated significantly [13, 14, 15]. Furthermore, high GDF‐15 levels are associated with reduced muscle mass and strength, frailty, slower walking speeds and diminished physical function in older adults [16, 17, 18]. Studies also report significant associations between elevated GDF‐15 levels and an increased risk of CKD progression, CVD and mortality in individuals with type 2 diabetes [19, 20, 21, 22, 23].

Despite these findings, the interplay between the five indices derived from cystatin C and creatinine and GDF‐15 remains poorly understood, particularly in individuals with diabetes mellitus. Moreover, the extent to which these markers are independently associated with adverse outcomes, such as CKD progression and mortality, has not been evaluated comprehensively. Therefore, we investigated whether the five indices, as well as GDF‐15, independently associate with adverse outcomes—including CKD progression (≥ 30% decline in eGFRcr from baseline), mortality and their composite outcome—after adjusting for baseline eGFRcr and the urinary albumin‐to‐creatinine ratio (UACR) in Japanese individuals with diabetes mellitus.

## 2 Methods

### 2.1 Study Design and Subjects

Individuals with diabetes were recruited from Kure Medical Center and Chugoku Cancer Center (Hiroshima, Japan) between 1 July 2014 and 31 March 2016 to observe the natural course of diabetic kidney disease (DKD). Of the 738 participants initially enrolled, 100 were excluded, primarily because of a baseline eGFR of < 30 mL/min/1.73 m^2^ or a follow‐up period of < 6 months. The final study population comprised 638 participants, as illustrated in Supplementary Figure S1.

The study was approved by the ethics committee of Kure Medical Center and Chugoku Cancer Center (approval number: 26‐06) and was conducted in accordance with the principles of the Declaration of Helsinki and the Ethical Guidelines on Clinical Studies established by the Ministry of Health, Labour and Welfare of Japan. Written informed consent was obtained from all participants.

### 2.2 Sample Collection and Laboratory Measurements

Blood samples were collected at registration from enrolled participants via venipuncture. After centrifugation at 2300 × *g* for 4 min at room temperature (Kubota S‐500T centrifuge; Kubota Co. Ltd., Tokyo, Japan), the serum fraction was transferred to 1.5 mL Eppendorf Safe‐Lock Tubes (Eppendorf SE, Hamburg, Germany) and stored at −80°C until subsequent assays. Serum creatinine and cystatin C levels were measured using a standard enzymatic method and a colloidal gold immunoassay [Nescauto GC Cystatin C (Nm); Alfresa Pharma Corp., Osaka, Japan], respectively.

eGFR was calculated using formulas specific to the Japanese population, following the Japanese Society of Nephrology guidelines [24, 25]:

- eGFRcr (mL/min/1.73 m^2^) = 194 × [age (years)]^−0.287^ × [serum creatinine (mg/dL)]^−1.094^ (× 0.739 for women)
- eGFRcys (mL/min/1.73 m^2^) = [104 × (serum cystatin C [mg/dL])^−1.019^ × 0.996^Age^ (× 0.929 for women)] − 8

Urinary albumin and creatinine levels were quantified using immunonephelometry (N‐assay TIA Micro Alb; Nittobo Medical Co. Ltd., Fukushima, Japan) and enzymatic methods, respectively. The UACR was expressed as mg of albumin per g of creatinine. Serum GDF‐15 levels were measured using an enzyme‐linked immunosorbent assay (cat. No. DGD150; R&D Systems, Minneapolis, MN, USA). Two internal serum controls were included in each assay for quality control purposes. The inter‐assay coefficient of variation for GDF‐15 was calculated to be 4.0%.

### 2.3 Five Indices Derived From Cystatin C and Creatinine That May Reflect Sarcopenic Status

Five indices potentially reflecting sarcopenia were calculated using formulas derived from serum creatinine and cystatin C levels [9]:

- eGFRdiff (mL/min/1.73 m^2^) = eGFRcys (mL/min/1.73 m^2^) − eGFRcr (mL/min/1.73 m^2^)
- SI = eGFRcys (mL/min/1.73 m^2^) × serum creatinine (mg/dL)
- CCR = serum creatinine (mg/dL)/serum cystatin C (mg/dL)
- pSMI = 4.17–0.012 × Age (y) + 1.24 × (serum creatinine [mg/dL]/serum cystatin C [mg/dL]) − 0.0513 × haemoglobin (g/dL) + 0.0598 × body weight (kg) (for men); 3.55–0.00765 × Age (y) + 0.852 × (serum creatinine [mg/dL]/serum cystatin C [mg/dL]) − 0.0627 × haemoglobin (g/dL) + 0.0614 × body weight (kg) (for women)
- TBMM = body weight (kg) × serum creatinine (mg/dL)/(k × body weight [kg] × serum cystatin C [mg/dL] + serum creatinine [mg/dL]) (the coefficient values [k] were 0.00675 for men and 0.01006 for women)

### 2.4 Muscle Mass Assessment

The muscle mass was estimated using appendicular skeletal muscle mass (ASM), calculated based on a previously validated equation for a Chinese population [26]:

- ASM = 0.193 × body weight (kg) + 0.107 × height (cm) − 4.157 × k − 0.037 × age (y) − 2.631 (the coefficient values [k] were 1 for men and 2 for women)

The skeletal muscle mass index (SMI) was then determined by dividing ASM by height squared in meters:

- SMI = ASM/height (m)^2^

Low muscle mass (LMM) was classified based on the 20th percentile cutoff of the study population. Low SMI was defined as < 7.19 kg/m^2^ for men and < 5.25 kg/m^2^ for women.

### 2.5 Adverse Outcomes (CKD Progression, Mortality and Composite Outcome)

CKD progression was defined as a 30% decline in eGFRcr from baseline during the follow‐up period, regardless of the baseline eGFRcr value. Participants were not required to meet the diagnostic criteria for CKD (i.e., eGFR < 60 mL/min/1.73 m^2^) at baseline. A 30% decline was used as the primary endpoint because only 37 (5.8%) patients experienced a 40% decline in eGFRcr from baseline by the end of the follow‐up period. Eligible participants were monitored every few months with blood tests. The number of eGFRcr assessments varied among participants; the mean number of measurements per participant during the study period was 33 (standard deviation [SD], 16).

The first occurrence of a 30% decline in eGFRcr from baseline, confirmed by two consecutive blood tests taken at least one month apart, was recorded as the onset of the CKD progression. Composite outcome was defined as either CKD progression (30% decline in eGFRcr from baseline) or mortality.

### 2.6 Statistical Analysis

Continuous variables are expressed as means ± SD for normal distribution or as medians with interquartile ranges (IQRs) for skewed variables, whereas categorical variables are presented as numbers and percentages. The normality of continuous variables was evaluated using a combination of statistical tests and graphical methods. Specifically, the Kolmogorov–Smirnov test was performed, and skewness and kurtosis were examined. Furthermore, histogram visualization was used to assess the overall distribution pattern.

Group comparisons were conducted using the Student's *t*‐test or Mann–Whitney U test for two groups and the one‐way analysis of variance or Kruskal–Wallis test for three groups, as appropriate. Categorical variables were analysed using the *χ* ^2^ test. Spearman's correlation analysis was used to assess relationships among GDF‐15, the five indices derived from cystatin C and creatinine (eGFRdiff, SI, CCR, pSMI, TBMM) and kidney function (UACR, eGFRcr, eGFRcys). Multiple‐ordered logistic regression was used to examine associations between baseline GDF‐15 tertiles and eGFRdiff, with GDF‐15 as the dependent variable and eGFRdiff as the independent variable, adjusting for age, sex, body mass index (BMI), haemoglobin, glycated haemoglobin, systolic blood pressure, uric acid, C‐reactive protein (CRP), UACR and eGFRcr or eGFRcys. Cox proportional hazards models with restricted cubic splines (spline knots = 4) were used to assess associations between GDF‐15, LMM, and the five indices (independent variables) with CKD progression, mortality and their composite outcome (dependent variables), adjusted for age, sex, glycated albumin, eGFRcr and UACR. Continuous variables, including GDF‐15 and the five indices, were treated as continuous and categorical for subgroup analyses.

All analyses were performed using SAS v.9.4 (SAS Institute Inc., Cary, NC, USA) and R version 4.4.2 (The R Foundation for Statistical Computing, Vienna, Austria), with a two‐tailed *p*‐value < 0.05 indicating statistical significance.

## 3 Results

### 3.1 Baseline Characteristics

In this study, approximately one in four participants (*n* = 163, 25.5%) had eGFRcys values lower than eGFRcr. Additionally, 53 participants (8.3%) exhibited an eGFRdiff of less than −15 mL/min/1.73 m^2^. The mean eGFRdiff was 14.3 ± 22.4 mL/min/1.73 m^2^. Participants were stratified into tertiles based on eGFRdiff values, and their clinical characteristics are summarized in Table 1. Individuals with higher eGFRdiff values demonstrated a higher male‐to‐female ratio and exhibited elevated levels of haemoglobin, uric acid and eGFRcys while having a lower BMI, glycated haemoglobin, CRP, eGFRcr and UACR.

**TABLE 1:** Baseline characteristics according to eGFRdiff levels categorized as tertiles in individuals with diabetes mellitus.

Characteristics		eGFRdiff (*n* = 638)		*p*
	Tertile 1 (*n* = 213) < 3.8	Tertile 2 (*n* = 213) 3.8–24.3	Tertile 3 (*n* = 212) > 24.3	
eGFRdiff, mL/min/1.73 m^2^	−9.5 ± 12.6	13.9 ± 6.4	38.7 ± 12.0	< 0.001
Age, year	66 (55, 73)	68 (60, 74)	67 (60, 71)	0.12
Male, *n* (%)	23 (10.8)	119 (55.9)	202 (95.3)	< 0.001
BMI, kg/m^2^	25.2 (22.1, 29.1)	24.1 (21.5, 27.0)	23.8 (21.7, 26.4)	< 0.001
Systolic BP, mmHg	140 ± 17	139 ± 19	136 ± 16	0.09
Diastolic BP, mmHg	78 ± 12	77 ± 12	79 ± 11	0.05
Haemoglobin, g/dL	13.0 ± 1.5	13.4 ± 1.8	14.4 ± 1.5	< 0.001
Glycated haemoglobin, %	7.4 (6.7, 8.1)	7.1 (6.6, 7.9)	7.1 (6.5, 7.7)	0.02
Type 2 diabetes, *n* (%)	195 (91.5)	188 (88.3)	185 (87.3)	0.34
HDL‐C, mg/dL	51 (45, 61)	50 (42, 60)	52 (45, 62)	0.30
Non‐HDL‐C, mg/dL	125 (108, 156)	126 (109, 150)	125 (108, 145)	0.65
Prior CVD, *n* (%)	21 (9.9)	29 (13.6)	26 (12.3)	0.48
eGFRcr, mL/min/1.73 m^2^	80 ± 27	67 ± 21	69 ± 17	< 0.001
eGFRcys, mL/min/1.73 m^2^	70 ± 23	81 ± 22	108 ± 21	< 0.001
UACR, mg/g	26 (12, 129)	20 (8, 130)	17 (7, 74)	0.004
Uric acid, mg/dL	5.1 ± 1.3	5.3 ± 1.5	5.4 ± 1.2	0.008
C‐reactive protein, mg/dL	0.12 (0.06, 0.19)	0.11 (0.06, 0.21)	0.07 (0.05, 0.13)	< 0.001
GDF‐15, pg/mL	1478 (1001, 2151)	1526 (984, 2140)	1334 (926, 1786)	0.005
SI	42.6 (38.2, 46.0)	65.9 (51.8, 76.1)	91.9 (85.9, 98.5)	< 0.001
CCR	0.64 ± 0.08	0.80 ± 0.08	0.98 ± 0.13	< 0.001
pSMI	6.6 ± 1.0	6.8 ± 0.9	7.1 ± 0.8	< 0.001
TBMM	31.7 ± 5.3	38.6 ± 7.3	45.1 ± 6.3	< 0.001
LMM, *n* (%)	38 (17.8)	46 (21.6)	44 (20.8)	0.60

*Note:* Data are presented as mean ± SD, medians (25th and 75th percentile) or *n* (percentage).

Abbreviations: BMI, body mass index; BP, blood pressure; CCR, serum creatinine to cystatin C ratio; CVD, cardiovascular disease; eGFR, estimated glomerular filtration rate; eGFRcr, serum creatinine‐based eGFR; eGFRcys, serum cystatin C‐based eGFR; eGFRdiff, difference between eGFRcys and eGFRcr; GDF‐15, growth differentiation factor 15; HDL‐C, high density cholesterol; LMM, low muscle mass; pSMI, prediction equation for skeletal mass index; SD, standard deviation; SI, sarcopenia index; TBMM, total body muscle mass index; UACR, urinary albumin to creatinine ratio.

Regarding the indices derived from cystatin C and creatinine, individuals with higher eGFRdiff values exhibited elevated SI, CCR, pSMI and TBMM. Notably, all five indices were significantly higher in males than in females, as shown in Supplementary Table S1.

### 3.2 Correlation Among GDF‐15, Indices Derived From Cystatin C and Creatinine and Kidney Function Markers

Spearman's correlation analysis revealed minimal correlations between GDF‐15 and the five indices derived from cystatin C and creatinine, with only a slight negative correlation observed (Supplementary Table S2). Given the significant sex differences in the five indices, a correlation analysis adjusted for age and sex was conducted. As shown in Table 2, age‐ and sex‐adjusted Spearman's correlation analysis indicated weak negative correlations between GDF‐15 and indices derived from cystatin C and creatinine (excluding pSMI), with correlation coefficients (*r*‐values) ranging from −0.12 (TBMM) to −0.35 (eGFRdiff). All pairwise comparisons among eGFRdiff, SI, and CCR showed extremely strong positive correlations, with *r*‐values ranging from 0.93 to 0.96. eGFRdiff showed a weak positive correlation with TBMM (*r* = 0.35) but no significant correlation with pSMI (*r* = 0.003).

**TABLE 2:** Age‐ and sex‐adjusted Spearman's correlation coefficients between GDF‐15, indices derived from cystatin C and creatinine, and kidney function markers.

Variables	eGFRdiff	SI	CCR	pSMI	TBMM	eGFRcr	eGFRcys	UACR
GDF‐15	−0.35*	−0.34*	−0.29*	0.03	−0.12**	−0.26*	−0.45*	0.28*
eGFRdiff	—	0.93*	0.93*	0.003	0.35*	−0.14*	0.54*	−0.23*
SI		—	0.96*	0.04	0.39*	−0.21*	0.48*	−0.21*
CCR			—	0.04	0.40*	−0.34*	0.35*	−0.16*
pSMI				—	0.87*	−0.14**	−0.08	0.10†
TBMM					—	−0.21*	0.10‡	0.02
eGFRcr						—	0.69*	−0.17*
eGFRcys							—	−0.28*

*Note:* Abbreviations used in this table are the same as in Table 1.

* *p* < 0.001.

** *p* < 0.005.

‡ *p* < 0.01.

† *p* < 0.05.

GDF‐15 demonstrated a moderate negative correlation with eGFRcys (*r* = −0.45) and a weak positive correlation with UACR (*r* = 0.28). Conversely, eGFRdiff exhibited a moderate positive correlation with eGFRcys (*r* = 0.54) and a weak negative correlation with UACR (*r* = −0.23). GDF‐15 (*r* = −0.26) and eGFRdiff (*r* = −0.14) showed weak negative correlations with eGFRcr.

### 3.3 Association Between GDF‐15 and eGFRdiff in Ordered Logistic Regression Analysis

As shown in Table 3, multiple‐ordered logistic regression analysis revealed that individuals with higher eGFRdiff values had significantly lower odds ratios (ORs) for increasing tertile levels of GDF‐15. This association persisted even after adjusting for age, sex, BMI, glycated haemoglobin, systolic blood pressure, uric acid, CRP, UACR and eGFRcr (OR 0.67, 95% confidence interval [CI]: 0.60–0.75, *p* < 0.001). A similar trend was observed when using eGFRcys instead of eGFRcr in the model, although the association between GDF‐15 and eGFRdiff was attenuated (OR 0.87, 95% CI: 0.76–0.97, *p* = 0.01).

**TABLE 3:** Univariate and multivariate ordered logistic regression analysis of eGFRdiff influencing circulating GDF‐15 levels.

	eGFRdiff OR (95% CI)	*p*
Model 1	0.91 (0.85, 0.97)	0.004
Model 2	0.67 (0.61, 0.75)	< 0.001
Model 3	0.73 (0.66, 0.82)	< 0.001
Model 4	0.67 (0.60, 0.75)	< 0.001
Model 5	0.86 (0.76, 0.97)	0.01

*Note:* Abbreviations used in this table are the same as in Table 1. Model 1, Unadjusted; Model 2, Age and sex; Model 3, Model 2 + BMI, haemoglobin, glycated haemoglobin, systolic BP, uric acid, C‐reactive protein, and UACR; Model 4, Model 3 + eGFRcr; Model 5, Model 3 + eGFRcys.

Abbreviations: CI, confidence interval; OR, odds ratio.

### 3.4 Association of GDF‐15 and eGFRdiff With Clinical Outcomes—Univariate Analysis

**FIGURE 1:**
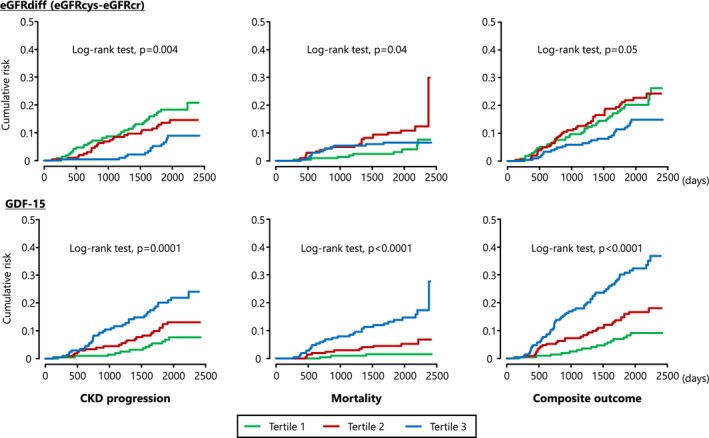
Cumulative risk of CKD progression, mortality and composite outcome in individuals with diabetes mellitus, according to the tertile of eGFRdiff and serum GDF‐15 at baseline. Abbreviations: CKD, chronic kidney disease; eGFR, estimated glomerular filtration rate; eGFRcr, serum creatinine‐based eGFR; eGFRcys, serum cystatin C‐based eGFR; eGFRdiff, difference between eGFRcys and eGFRcr; GDF‐15, growth differentiation factor 15.

The median follow‐up was 5.3 years (IQRs: 3.7–6.0 years) for CKD progression, 5.4 years (4.8–6.0) for death and 5.3 years (3.8–6.0) for the composite outcome. During this period, 75 participants (11.8%) experienced CKD progression, 44 (6.9%) died from all causes and 112 (17.6%) reached the composite outcome. The cumulative incidence of adverse outcomes was stratified by tertiles of eGFRdiff and GDF‐15 (Figure 1). For eGFRdiff, the cumulative incidence of CKD progression increased progressively as eGFRdiff declined from the highest to the lowest tertile. For GDF‐15, the cumulative incidence of adverse outcomes (CKD progression, mortality and composite outcome) increased progressively from the lowest to the highest tertile over time.

### 3.5 Association of GDF‐15, LMM and Indices Derived From Cystatin C and Creatinine With Clinical Outcomes—Multivariate Analysis

As shown in Table 4, individuals in the highest eGFRdiff tertile (T3) had lower adjusted HRs compared with those in the lowest tertile (T1), as follows: 0.21 (95% CI: 0.09–0.48) for CKD progression, 0.49 (95% CI: 0.16–1.49) for mortality and 0.28 (95% CI: 0.14–0.54) for composite outcome. Each 10‐unit increase in eGFRdiff was associated with a 33% reduction in the risk of CKD progression (hazard ratio [HR]: 0.67), a 20% reduction in the risk of mortality (HR: 0.80) and a 28% reduction in the risk of composite outcome (HR: 0.72). Conversely, individuals in the highest GDF‐15 tertile (T3) had higher adjusted HRs compared with those in the lowest tertile (T1), as follows: 1.61 (95% CI: 0.79–2.37) for CKD progression, 5.47 (95% CI: 1.59–18.80) for mortality and 2.37 (95% CI: 1.30–4.30) for composite outcome. Each one SD increase in logarithm‐transformed GDF‐15 was associated with a 36% higher risk of CKD progression (HR: 1.36), a 235% higher risk of mortality (HR: 2.35) and a 62% higher risk of composite outcome (HR: 1.62).

**TABLE 4:** Association of GDF‐15 or eGFRdiff with CKD progression, mortality and composite outcome.

	GDF‐15^a^ (per 1SD increase) HR (95% CI)	T1	T2 HR (95% CI)	T3 HR (95% CI)	eGFRdiff (per 10 increase) HR (95% CI)	T1	T2 HR (95% CI)	T3 HR (95% CI)
CKD progression								
Model 1	1.85 (1.44, 2.38)*	ref.	1.86 (0.95, 3.65)	3.48 (1.85, 6.54)**	0.82 (0.74, 0.91)*	ref.	0.73 (0.44, 1.21)	0.37 (0.20, 0.69)‡
Model 2	1.87 (1.44, 2.41)*	ref.	1.84 (0.91, 3.73)	3.45 (1.79, 6.66)**	0.70 (0.63, 0.79)*	ref.	0.44 (0.24, 0.80)‡	0.16 (0.08, 0.35)*
Model 3	1.36 (1.02, 1.81)†	ref.	1.41 (0.70, 2.86)	1.61 (0.79, 2.37)	0.67 (0.58, 0.78)*	ref.	0.42 (0.21, 0.83)†	0.21 (0.09, 0.48)**
Mortality								
Model 1	2.70 (1.95, 3.73)*	ref.	3.51 (0.98, 12.60)	10.35 (3.16, 34.00)**	1.03 (0.91, 1.18)	ref.	2.58 (1.19, 5.61)†	1.45 (0.62, 3.40)
Model 2	2.35 (1.66, 3.33)*	ref.	2.04 (0.56, 7.41)	5.69 (1.70, 19.07)‡	0.80 (0.79, 0.82)†	ref.	1.05 (0.41, 2.69)	0.48 (0.16, 1.42)
Model 3	2.35 (1.63, 3.41)*	ref.	2.01 (0.55, 7.29)	5.47 (1.59, 18.80)‡	0.80 (0.65, 0.99)†	ref.	1.00 (0.38, 2.66)	0.49 (0.16, 1.49)
Composite outcome								
Model 1	2.07 (1.69, 2.54)*	ref.	2.06 (1.13, 3.74)†	4.59 (2.65, 7.95)*	0.90 (0.83, 0.97)‡	ref.	1.07 (0.70, 1.63)	0.61 (0.38, 0.99)†
Model 2	1.97 (1.59, 2.44)*	ref.	1.63 (0.88, 3.02)	3.66 (2.07, 6.46)*	0.73 (0.66, 0.81)*	ref.	0.57 (0.34, 0.96)†	0.24 (0.13, 0.45)*
Model 3	1.62 (1.28, 2.06)*	ref.	1.42 (0.76, 2.62)	2.37 (1.30, 4.30)‡	0.72 (0.64, 0.81)*	ref.	0.53 (0.30, 0.93)†	0.28 (0.14, 0.54)**

*Note:* Abbreviations used in this table are the same as in Tables 1 and 3. Model 1, unadjusted; Model 2, age and sex; Model 3, Model 2 + glycated haemoglobin, UACR, and eGFRcr.

Abbreviations: CKD, chronic kidney disease; HR, hazard ratio; T, tertile.

^a^ 1SD (0.24) increase of logarithm‐transformed GDF‐15.

* *p* < 0.0001.

** *p* < 0.001.

‡ *p* < 0.01.

† *p* < 0.05.

Other indices (SI, CCR, TBMM and LMM), except for pSMI, were associated with CKD progression and composite outcome (Supplementary Table S3). Additionally, SI and CCR were associated with mortality (Supplementary Table S3).

### 3.6 Impact of eGFRdiff and GDF‐15 on Outcomes Assessed by Cox Proportional Hazards Models With Restricted Cubic Splines

**FIGURE 2:**
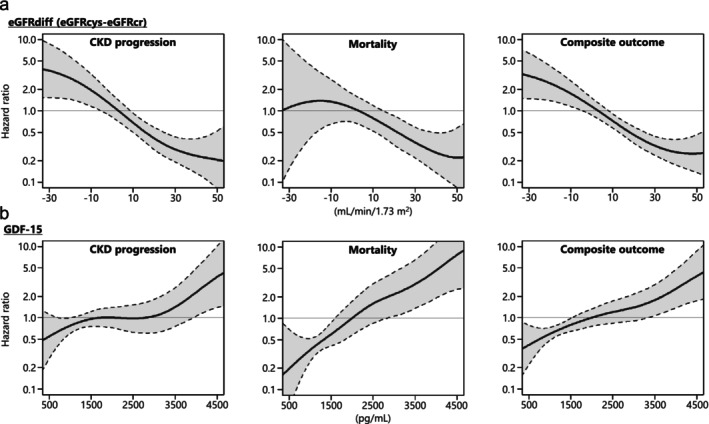
Hazard ratios for the development of CKD progression, mortality and composite outcome by (a) eGFRdiff and (b) GDF‐15 at baseline in multivariate Cox proportional hazard model analysis with a restricted cubic spline after adjustment of age, sex, glycated haemoglobin, UACR and eGFRcr. Solid line: HR; dashed line: 95% confidence interval. The reference value of eGFRdiff was 3 mL/min/1.73 m^2^ and GDF‐15 was 2000 pg/mL. Abbreviations: CKD, chronic kidney disease; eGFR, estimated glomerular filtration rate; eGFRcr, serum creatinine‐based eGFR; eGFRcys, serum cystatin C‐based eGFR; eGFRdiff, difference between eGFRcys and eGFRcr; GDF‐15, growth differentiation factor 15.

For CKD progression, the cubic spline curve for eGFRdiff demonstrated a gradual decline in risk as eGFRdiff increased (Figure 2a). In contrast, the cubic spline curve for GDF‐15 exhibited a biphasic pattern, with an initial slow ascent, a plateau phase and a subsequent sharp rise as GDF‐15 levels increased (Figure 2b).

For mortality, the cubic spline curve for eGFRdiff declined with higher values but plateaued when eGFRdiff reached approximately −10 mL/min/1.73 m^2^ (Figure 2a). In contrast, the relationship between GDF‐15 and mortality appeared monotonic, with risks rising steadily as GDF‐15 levels increased (Figure 2b).

## 4 Discussion

In this observational cohort study of Japanese individuals with diabetes mellitus, we identified for the first time that lower eGFRdiff is consistently associated with a higher risk of adverse outcomes, including CKD progression, mortality and composite outcome. Additionally, higher GDF‐15 levels were associated with these adverse outcomes. Although both markers were identified as risk factors, eGFRdiff showed a stronger association with CKD progression, whereas GDF‐15 was more strongly associated with mortality.

eGFRdiff has been reported to be influenced by factors beyond intrinsic kidney function. In our study, participants in the lowest eGFRdiff tertile (T1) had the highest eGFRcr and the lowest eGFRcys, potentially reflecting reduced muscle mass and low serum creatinine levels. Furthermore, eGFRdiff was strongly correlated with other indices derived from cystatin C and creatinine, including SI and CCR, and weakly correlated with TBMM. When these indices were used instead of eGFRdiff, all except for pSMI showed significant associations with CKD progression. Importantly, eGFRdiff remained significantly associated with GDF‐15, a marker increasingly linked to sarcopenia, even after adjusting for eGFRcr or eGFRcys. These findings suggest that lower eGFRdiff may, at least in part, reflect sarcopenia.

According to Grubb et al. [27], an eGFRcys/eGFRcr ratio below 0.8 (equivalent to an eGFRdiff of less than −20%) may indicate ‘shrunken pore syndrome’, an early manifestation of CKD characterized by selective glomerular filtration impairment of medium‐sized molecules (10 000–20 000 Da). Although this study did not fully elucidate the mechanisms underlying lower eGFRdiff values, our findings suggest that these values may be associated with adverse outcomes beyond what is captured by eGFR alone.

Previous studies in US and Swedish cohorts have linked lower eGFRdiff with an increased risk of kidney function decline, progression to end‐stage kidney disease, acute kidney injury and mortality [8, 28]. In a UK diabetes cohort, lower eGFRdiff values were associated with incident DKD, even in the absence of microvascular complications [29]. Unlike the UK study, which used ICD‐10 diagnostic codes to define outcomes, our study adds value by clearly defining CKD progression as a ≥ 30% decline in GFR from baseline.

In Western cohorts with hypertension, advanced age and CKD, the prevalence of eGFRdiff below −15 mL/min/1.73 m^2^ ranged from 8% to 16% [5, 7, 30]. In the UK diabetes cohort, the prevalence was approximately 40%, significantly higher than previous reports [29]. However, in our study, only 8.3% of participants demonstrated eGFRdiff below this threshold, even in the diabetes cohort. These differences may stem from variations in eGFR estimation equations between Western countries and Japan or the lower incidence of adverse outcomes in Japanese populations [31]. A Japanese community‐based study of older adults reported median eGFRdiff, CCR, SI, pSMI and TBMM levels differing from those in our cohort [9]. These discrepancies may reflect demographic and clinical differences. Nevertheless, our study demonstrates that the association between eGFRdiff and adverse outcomes persists even after adjusting for eGFRcr, underscoring the potential influence of non‐GFR determinants.

The reason GDF‐15 is more strongly associated with mortality than CKD progression, compared with eGFRdiff, remains unclear. However, the involvement of GDF‐15 in various pathophysiological processes leading to sarcopenia may partly contribute to this association. For example, GDF‐15 overexpression in genetically modified mice or the administration of GDF‐15 to diabetic model mice or GDF‐15 knockout mice results in weight loss [32, 33, 34]. Previous studies have shown that GDF‐15 promotes anorexia through its receptor, glial cell line‐derived neurotrophic factor family receptor α‐like, and the co‐receptor rearranged during transfection [35]. Thus, GDF‐15 may be strongly associated with mortality because of its significant involvement in weight loss, which affects energy metabolism.

This study has several limitations. First, causality cannot be inferred because of the observational design. Second, the number of adverse events was insufficient to allow comprehensive adjustment for potential confounders. Third, indices derived from cystatin C and creatinine were assessed at a single baseline measurement, raising the possibility of misclassification. Fourth, as the study population consisted of Japanese individuals with diabetes mellitus, the findings may not be generalizable to ethnically diverse populations. Fifth, although sodium‐glucose cotransporter‐2 (SGLT2) inhibitors and glucagon‐like peptide‐1 (GLP‐1) receptor agonists have been shown to provide cardiovascular and renal benefits [36, 37, 38], their influence on our findings is likely minimal. Only 1.7% of participants were receiving an SGLT2 inhibitor, and 5.0% were on a GLP‐1 receptor agonist. Moreover, most participants were enrolled before these agents became widely recognized for their protective effects. As a result, while we acknowledge the potential impact of these medications, their effect on our results is expected to be limited. Future studies with a larger patient cohort receiving these agents are needed to further validate our findings. Sixth, serum levels of GDF‐15 are elevated in various types of cancer. However, the exact proportion of patients with cancer in this study population is not clearly specified. Seventh, muscle mass was not directly measured using imaging techniques or bioelectrical impedance analysis, but was estimated indirectly. This approach may have led to inaccuracies in assessing the influence of muscle mass on eGFR differences, particularly in individuals with abnormal body composition. Finally, while eGFRdiff and other indices derived from cystatin C and creatinine were associated with adverse outcomes, the absence of sarcopenia diagnosis based on established criteria limits conclusions about the relationship between these indices and sarcopenia.

In conclusion, this observational study of individuals with diabetes mellitus revealed two key findings: (1) eGFRdiff was associated with GDF‐15 independent of eGFRcr and (2) both eGFRdiff and GDF‐15 were associated with the risk of CKD progression, mortality and their composite outcome. These results suggest that frailty and sarcopenia, driven by inflammation and metabolic abnormalities, may contribute to CKD progression and mortality in individuals with diabetes mellitus.

Furthermore, approximately one‐quarter of participants exhibited a negative difference between eGFRcys and eGFRcr (eGFRdiff). Given its ability to independently associate with adverse outcomes beyond eGFRcr, the combined assessment of eGFRcys and eGFRcr may enhance the identification of high‐risk individuals with diabetes mellitus. Future research should focus on identifying modifiable factors that contribute to lower eGFRdiff values and elucidating their clinical implications.

## Conflicts of Interest

The authors declare no conflicts of interest.

## Supporting information


**Figure S1.** Selection of study participants.

**Table S1.** Baseline characteristics stratified by sex in individuals with diabetes mellitus.
**Table S2.** Spearman's correlation coefficients between GDF‐15, indices derived from cystatin C and creatinine, and kidney function markers.
**Table S3.** Association of LMM and indices derived from cystatin C and creatinine with CKD progression, mortality and composite outcome.
