# Supplementary material for: Association of Difference Between eGFR From Cystatin C and Creatinine and Serum GDF‐15 With Adverse Outcomes in Diabetes Mellitus
Source: J Cachexia Sarcopenia Muscle. 2025 Jul 23;16(4):e70011. doi: 10.1002/jcsm.70011 (PMC12285683; doi:10.1002/jcsm.70011)
Supplement: Supplementary file 1 — Figure S1. Selection of study participants. [file JCSM-16-e70011-s002.pptx]

## Slide 1
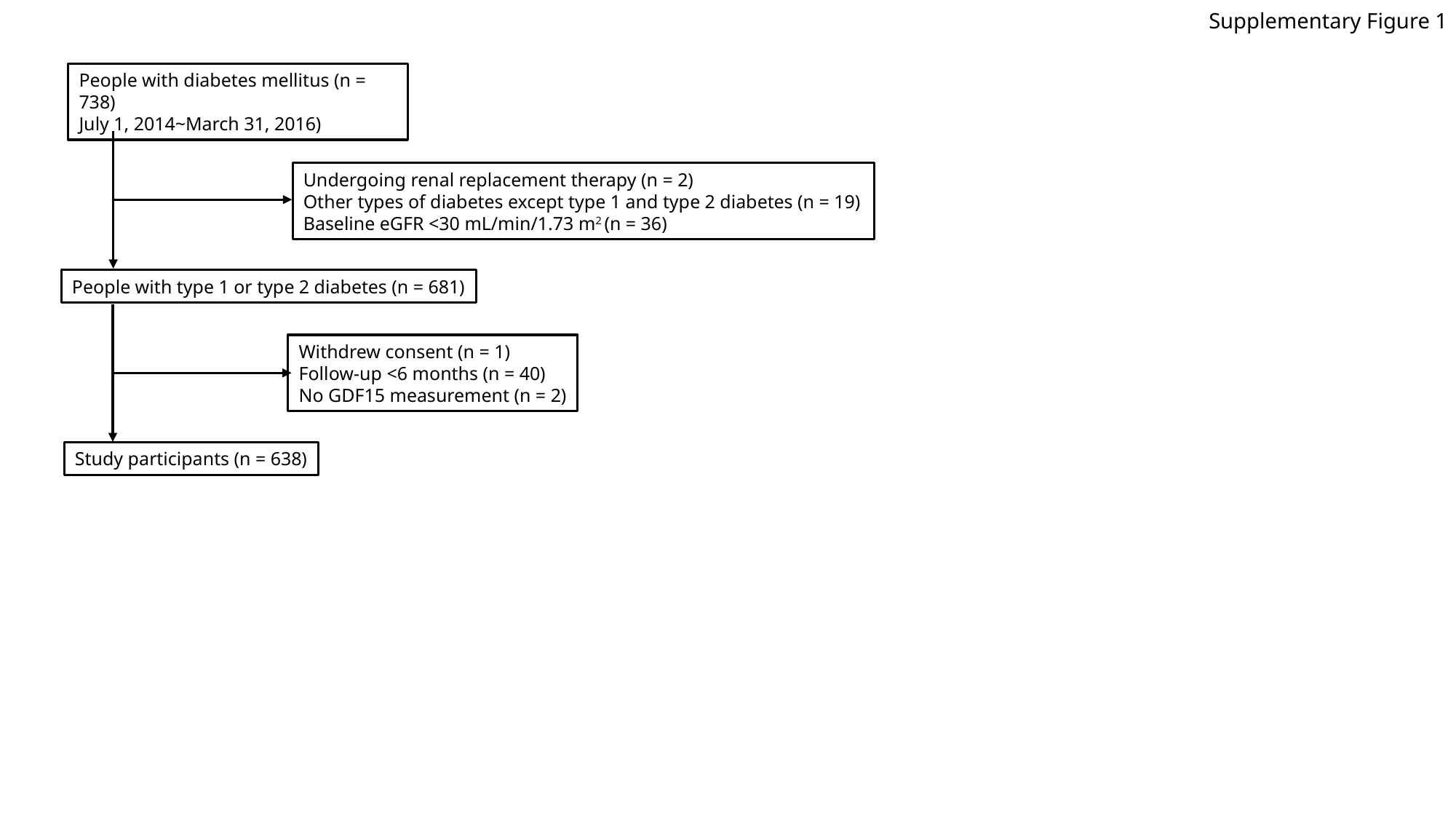

Supplementary Figure 1
People with diabetes mellitus (n = 738)
July 1, 2014~March 31, 2016)
Undergoing renal replacement therapy (n = 2)
Other types of diabetes except type 1 and type 2 diabetes (n = 19)
Baseline eGFR <30 mL/min/1.73 m2 (n = 36)
People with type 1 or type 2 diabetes (n = 681)
Withdrew consent (n = 1)
Follow-up <6 months (n = 40)
No GDF15 measurement (n = 2)
Study participants (n = 638)
